# Involvement of testicular N-glycoproteome heterogeneity in seasonal spermatogenesis of the American mink (*Neovison vison*)

**DOI:** 10.3389/fvets.2022.1001431

**Published:** 2022-11-04

**Authors:** Yufei Zhang, Baozeng Xu

**Affiliations:** Key Laboratory for Molecular Biology of Special Economic Animals, Institute of Special Animal and Plant Sciences, Chinese Academy of Agricultural Sciences, Changchun, China

**Keywords:** seasonal spermatogenesis, N-glycosylation, proteomic profiles, American mink, testis

## Abstract

Spermatogenesis in the American mink is characterized by an annual cycle of transition involving completely inactive and fully activated stages. N-glycosylation of proteins has emerged as an important regulator as it affects protein folding, secretion, degradation, and activity. However, the function of protein N-glycosylation in seasonal spermatogenesis of the American mink remains unclear. In the present study, we established a proteome-wide stoichiometry of N-glycosylation in mink testes at various phases of spermatogenesis using N-linked glycosylated-peptide enrichment in combination with liquid chromatography-tandem mass spectrometry analysis. A total of 532 N-glycosylated sites matching the canonical Asn-X-Ser/Thr motif were identified in 357 testicular proteins. Both the number of glycoproteins and the sites of N-glycosylated proteins in mink testes were highly dynamic at different stages. Functional analyses showed that testicular proteins with different N-glycosylation might play a vital role in spermatogenesis by affecting their folding, distribution, stability, and activity. Overall, our data suggest that the dynamics of N-glycosylation of testicular proteins are involved in seasonal spermatogenesis in the American mink.

## Introduction

Mature spermatozoa are formed from primordial germ cells within the epithelium of the seminiferous tubules in testes during the process of mammalian spermatogenesis. During this period, biological events such as stem cell renewal and differentiation, mitotic and meiotic division, cell migration, nuclear shaping, and flagellum biogenesis occur sequentially (1, 2). Male germ cells reach mitotic arrest and do not start meiosis in the embryo until after birth. Unlike the female germ cells that spend years stuck in meiotic prophase-I, the male germ cell continues uninterruptedly through meiosis (3, 4). However, some animals have evolved many seasonal-specific behavioral and physiological adaptations that allow them to cope with and exploit the cyclical annual environment for reproduction. Mink is a typical seasonal breeder. Sexually mature male mink undergoes periodic spermatogenesis, degeneration and restart. Whereas the non-seasonal breeders such as mice, rats and humans, whose spermatogenesis occurs uninterruptedly throughout a lifetime after sexual maturity. Annual testicular activity in mink can be divided into three main phases: the period of maximum sexual activity (February–March), the period of sexual inactivity (May–October), and the onset of recovery of testicular activity (November) (4, 5). Notably, the biological events that occur during the initial phase of re-recovery after testicular regression in mink are similar or identical to those of spermatogenesis at the onset of sexual maturation in non-seasonal mammals. Therefore, mink can serve as an ideal animal model to study the molecular mechanisms of mammalian spermatogenesis (5).

It is generally known that post-translational modifications (PTMs) play an important role in the regulation of spermatogenesis (6). One of the significant PTMs in eukaryotes is the glycosylation of proteins, which regulates protein folding, quality control, sorting, degradation, and secretion, thereby ensuring the correct expression of key enzymes and structural proteins during spermatogenesis (7, 8). Protein glycosylation is highly dynamic and regulated by glycosyltransferases and glycosidases. These enzymes are responsible for adding different sugar residues to proteins and cleaving sugar residues from glycoproteins, respectively. N-linked glycosylation and O-linked glycosylation are the two currently well-known types of protein glycosylation. N-linked glycosylation takes place in the lumen of endoplasmic reticulum, transferring different sugar residues to nitrogen of protein asparagine or arginine residues. The transfer of various sugar residues to the hydroxy oxygen atom of serine, threonine, tyrosine, hydroxylysine, or hydroxyproline residues is known as O-linked glycosylation and occurs in the Golgi. Evidence demonstrates that both types of glycosylation are observed in proteins of epididymal fluid and are associated with spermatozoa (9, 10). However, little is known about the role of protein glycosylation potentially in mink seasonal spermatogenesis.

Besides serving as a biomedical research model for spermatogenesis, mink is a fur animal of extremely high economic value and is widely farmed in Nordic countries and northeastern China. The main challenge in fur farming has always been how to reproduce them efficiently. This study aimed to characterize how N-glycosylation modifications in testis proteins regulate spermatogenesis in mink, and lay the foundation for improving the reproductive efficiency of farmed mink.

## Materials and methods

### Animals

From the experimental base of the Chinese Academy of Agricultural Sciences in Jilin Province, 18 healthy, adult male minks (*Mustela vison*), weighing 2.0–2.5 kg, were raised in individual cages under circumstances of natural temperature and light. These animals received semi-annual canine distemper and parvovirus vaccinations to maintain their health. Farmworkers used physiological and behavioral indications to confirm the health of the animals, allowing sick animals to be quarantined. These mink were housed separately in cages outside and fed a wet diet of fish, chicken eggs, beef liver, extruded maize, mineral, and vitamin supplements (11). Water was available *ad libitum*.

### Sample collection

All animal experiments were conducted following the Guide to Care and Use of Experimental Animals issued by the Animal Ethics Committees of the Institute of Special Animal and Plant Sciences, Chinese Academy of Agricultural Sciences. Anesthesia was administered by intracardiac injection of 0.9 mL/kg body weight of phenobarbital sodium and 0.15 mL/kg of a solution of 0.3 g/ml chloral hydrate. The anesthetized mink were administered a lethal dose of succinylcholine chloride (Shanghai Xudong Haipu Pharmaceutical Co., Ltd., China) after sample collection. Testicular size and weight were measured immediately after collection. The right testis of each mink was snap frozen in liquid nitrogen immediately after collection for protein glycosylation and gene transcript level determination, while the other testis for other experiments. Mink testes were collected at the beginning of testicular activity recovery (November 15th, 2016; *n* = 6), mid-recovery (December 15th, 2016; *n* = 6) and pre-breeding season (February 15th, 2017; *n* = 6), respectively.

### Histomorphology staining

After being deparaffinized in xylene, the 5-μm slices were rehydrated using a gradient of ethanol to water concentrations. The slices were treated quickly with 1% hydrochloric acid dissolved in 75% ethanol and then subjected to 0.1% ammonia for about 10 sec after being stained with Harris hematoxylin for 20 min. The sections were counterstained for 5 min with 0.1% eosin and mounted in neutral balsam after being dried in 95% ethanol.

### Extraction and digestion of protein

The snap-frozen testis tissue was finely grounded in liquid nitrogen followed by solubilization in 1/10 volumes of SDT buffer (4% SDS, 100 mM DTT, and 150 mM Tris–HCl, pH 8.0) after evaporation of liquid nitrogen. After 5-min incubation in boiling water, the suspensions were sonicated (80 w, 10-sec ultrasonic bursts at a time for ten times). The crude extract was centrifugated at 14,000 g for 10-min at 4°C and the supernatant was carefully collected. The concentration of proteins was determined by the Bradford assay.

Protein digestion was performed following the filter-aided sample processing (FASP) procedure as described by Wisniewski, Zougman et al. (12). Through repeated ultrafiltration (Pall units, 10 kDa), the detergent, DTT, and other low-molecular-weight components were removed using UA buffer (8 M Urea, 150 mM Tris-HCl, pH 8.0). The proteins were alkylated before digestion by the addition of 100 μL IAA (50 mM IAA in UA) and incubated at room temperature for 30-min in the dark. After that, the samples were washed twice with 100 μL UA buffer and 100 μL DS buffer (50 mM triethylammonium bicarbonate, pH 8.5), respectively. Finally, the protein suspensions were digested overnight with 2 μg trypsin (Promega, USA) in a 40 μL DS buffer at 37°C, and the filtrates were collected for the resulting peptides. Peptide content was estimated by measuring absorbance at 280 nm (13).

### Glycopeptide enrichment

The collected peptides were mixed with 50 μL CWR (90 μg concanavalin A, 90 μg wheat germ agglutinin, and 90 μg *Ricinus* communis agglutinin) and incubated at room temperature for 1-h in a 30 kDa ultrafiltration tube. After centrifugation, the eluent was removed, and glycosylated peptides were captured and washed four times with 200 μL 1 ×BB buffer (2 mM CaCl_2_, 2 mM MnCl_2_, and 1 M NaCl in 40 mM Tris-HCl, pH 7.3). The unbound peptides were removed by the addition of 50 μL 25 mM NH_4_HCO_3_ prepared with ${\text{H}}_{2}^{18}$O and centrifugation. After deglycosylation at 37°C for 3 h with 2 μg PNGase-F in 50 μL 25 mM NH_4_HCO_3_ in ${\text{H}}_{2}^{18}$O, the captured peptides were washed with 40 μL 25 mM NH_4_HCO3 in ${\text{H}}_{2}^{18}$O and the filtrate collected by centrifugation.

### Liquid chromatography separation and mass spectrometric analysis

LC-MS/MS analysis was performed by EASY-μL C 1000 HPLC coupled with Q Exactive mass spectrometer (Thermo Fisher Scientific). The column was equilibrated with solution A (0.1% formic acid and 2% acetonitrile in MilliQ water). The samples were loaded using an autosampler on the peptide traps Thermo-Scientific EASY column (100 μm × 20 mm, 5.0 μm Aqua C18). Separation was carried out on a Thermo-Scientific EASY column (75 μm × 100 mm, 3 μm Aqua C18) at a flow rate of 300 μL/min with the following gradient: 0–45% solution B (0.1% formic acid and 84% acetonitrile in MilliQ water) for 0–115 min, 45–100% solution B for 115–118 min, and 100% solution B for 118–120 min. The mass spectrometer was operated in positive-ion mode with a data-dependent acquisition mode. Full scan MS spectra (from m/z 300 to 1800) were acquired with the Orbitrap at a high resolution of 70,000 (m/z 200). The automatic gain control target value was set to 3 × 10^6^, and dynamic exclusion was 25.0 s. Ten MS2 scans were captured after each full scan and fragmented by higher-energy collisional dissociation (HCD) with a normalized collision energy of 27 eV. Subsequently, MS/MS spectra were acquired using the Orbitrap with a resolution of 17,500 at m/z 200, the target value was 5 × 10^4^ with a maximum injection time of 60 ms, and the underfill ratio was set to 0.1%.

### Data analysis

The raw MS file was processed and searched using the MaxQuant software (version 1.3.0.5) against the UniProt Mustela database (39626 entries, downloaded on May 4, 2017) and the Decoy database. The following search parameters were used: Peptide Mass Tolerance = 10 ppm, Fragment Mass Tolerance = 0.1 Da, Enzyme = Trypsin, Missed cleavage = 2; Fixed modification: Carbamidomethyl (C), Variable modification: Oxidation (M), Deamidation^18^O (N), Peptide FDR ≤ 0.01, Site FDR ≤ 0.01.

### Bioinformatic analysis

The identified proteins were classified according to annotations from the UniProt database (http://www.uniprot.org/). The multi-omics data analysis tool, OmicsBean (http://www.omicsbean.cn), was used to analyze the differentially expressed protein (DEP), in which distributions in biological process (BP), cellular components (CCs), and molecular functions (MF) were assigned to each protein based on Gene Ontology (GO) categories. For pathway analysis, the differentially expressed proteins were mapped to the terms in the KEGG (Kyoto Encyclopedia of Genes and Genomes) database using OmicsBean. Protein-protein interaction networks were analyzed using the publicly available program STRING (http://string-db.org/), and the minimum required interaction score was set to 0.400.

### Reverse transcription-quantitative real-time PCR

Total RNA was extracted from the frozen testes for quantitative analysis using the RNeasy Mini Extraction Kit (Qiagen) according to the manufacturer's instructions. Using MMLV reverse transcriptase and random primers, RNA (2 μg) was reverse-transcribed into first-strand cDNA (Roche Diagnostics, USA). Real-time PCR was performed using the Light Cycler 480 system (Roche Diagnostics, USA) with SYBR Green PCR Master Mix. The primer sequences are listed in Supplementary Table S1. A housekeeping gene was the GAPDH gene. In brief, 45 cycles of 95 °C for 10 sec and 60°C for 20 sec were performed after the initial pre-incubation at 95°C for 10 min. To confirm that single PCR amplicons were formed in each amplification, the PCR melting curves were examined. Nucleotide sequencing was used to verify the product's identity. By assessing amplification efficiencies using calibration curves and confirming that the plot of log input quantity vs. ΔCq has a slope of |0.1|, the assay performance was confirmed. At least three different frozen testes from each group were tested, and each sample was assayed in triplicate. Quantitative variation and relative fold change were calculated based on the 2^−ΔΔCt^ method.

### Statistical analysis

Statistical analyses and graphing were performed using GraphPad Prism 8 (GraphPad Software, Inc., San Diego, CA). Data are expressed as the mean difference with 95% confidence intervals of at least three different experiments. Student's t-test or one-way ANOVA followed by Tukey's multiple comparison test was used to analyze the data. Differences at *p* < 0.05 were considered significant.

## Results

### Seasonal spermatogenesis in mink testis from November to February confirmed through histological examination

**Figure 1:**
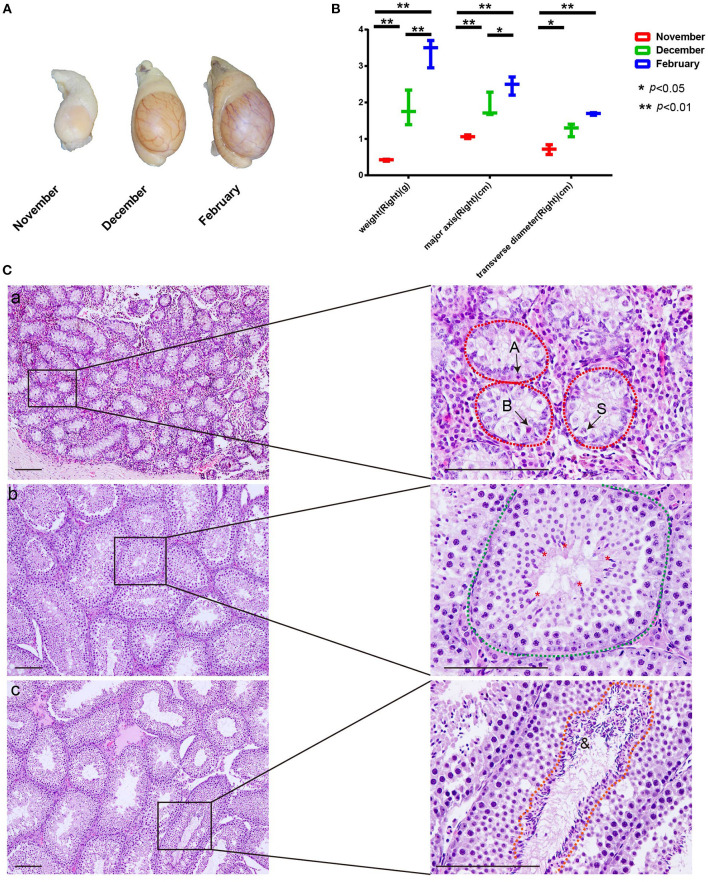
Changes in testis weight and histological morphology of seminiferous tubule during seasonal spermatogenesis in mink. **(A)** The volume of mink testes increased significantly during seasonal spermatogenesis. **(B)** Mink testis weight enhanced significantly with volume during seasonal. spermatogenesis. **(C)** In November, the seminiferous tubules were composed of large gonocyte-like germ cells (A: Type A spermatogonia, B: Type B spermatogonia, S: Sertoli cell). Furthermore, the Sertoli cell nuclei migrated to the center of the seminiferous tubules **(C-a)**. In December, spermatocyte meiosis was completed. Step 7 spermatids were present in the seminiferous epithelium (*: Spermatids) **(C-b)**. In February, the seminiferous tubules of the mink exhibited a normal spermatogenic epithelium during the breeding season (&: Sperm) **(C-c)**.

To test spermatogenesis onset following the sexual maturity of male mink during the transition from anestrus to estrous, we collected mink testes at different time points from November to February, during which the testicular development is triggered by increasing levels of testosterone hormone (14). As expected, the weight of the testis was 0.42 ± 0.02 g, 1.83 ± 0.39 g, and 3.4 ± 0.32 g in November, December, and February, respectively. In parallel, the volume of testes also increased from November to February (Figures 1A,B). For an accurate and reliable study of seasonal spermatogenesis, we examined the time course of development of the microscopic structure of seminiferous in mink. As shown in Figure 1C **(a)**, in addition, to type A1 spermatogonia, there were present also numerous type B spermatogonia derived from the spermatogonial stem cells distributed along the seminiferous tubule limiting membrane. Thus, mid-December was considered the beginning or re-initiation of the active spermatogenic phase in mink. Spermatocyte meiosis was finished by the middle of December, and step-7 spermatids appeared in the seminiferous epithelium (Figure 1C) **(b)**. Until mid-February, spermatogenesis had been completed to step-19 spermatids (Figure 1C), according to the definition of spermatid development proposed by R.-Marc Pelletier (14).

### Identification of total N-glycosylation sites and N-glycosylated proteins in mink testis during seasonal spermatogenesis

**Figure 2:**
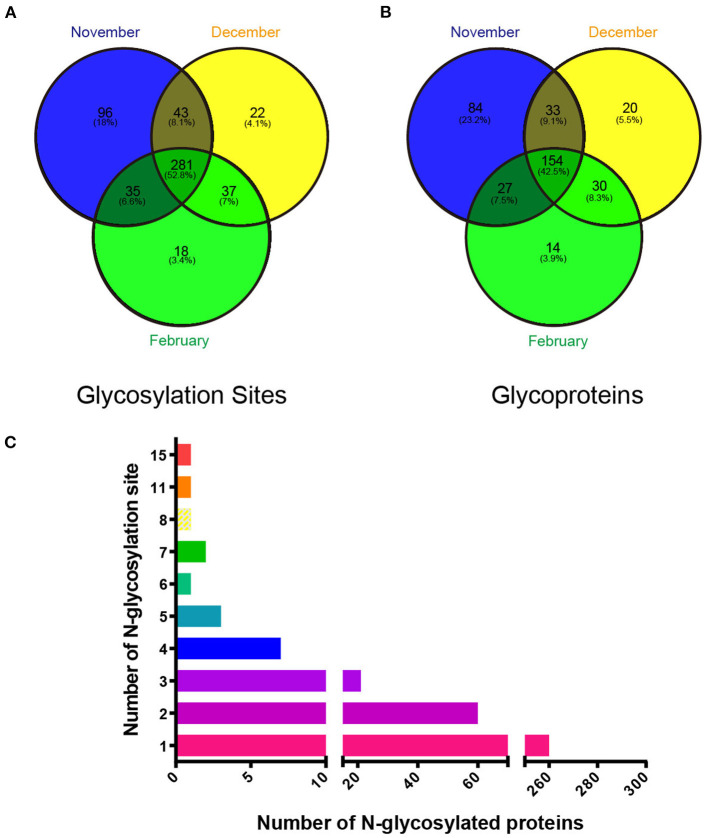
Identification of glycoproteins and their N-glycosylation sites in mink testes at different stages during spermatogenesis. **(A)** Changes in testis protein N-glycosylation sites at different stages. **(B)** Changes in testicular N-glycosylated proteins at different stages. **(C)** Changes in the number of N-glycosylation sites on different glycosylated proteins.

To test whether N-glycosylation is involved in seasonal spermatogenesis in mink, we evaluated the N-glycoproteome in testicular samples at different spermatogenesis stages using LC-MS/MS analysis following glycopeptide enrichment. A total of 532 glycosylation sites (Figure 2A), out of 357 glycosylated proteins (Figure 2B), were identified in all samples tested (Supplementary Table S2). Of these, 260 proteins contained a single N-glycosylation site, 60 proteins had two N-glycosylation sites, 31 proteins had three to five N-glycosylation sites, and six proteins with six or more N-glycosylation sites (Figure 2C). In particular, integrin beta, a non-covalently linked heterodimeric transmembrane receptor, was identified as having 11 N-glycosylation sites. Additionally, the current study discovered that low-density lipoprotein receptor-related protein 1 (LRP1) had up to 15 N-glycosylation sites.

### Characterization of the N-glycosylation sites and N-glycosylated proteins in mink testes during seasonal spermatogenesis

**Figure 3:**
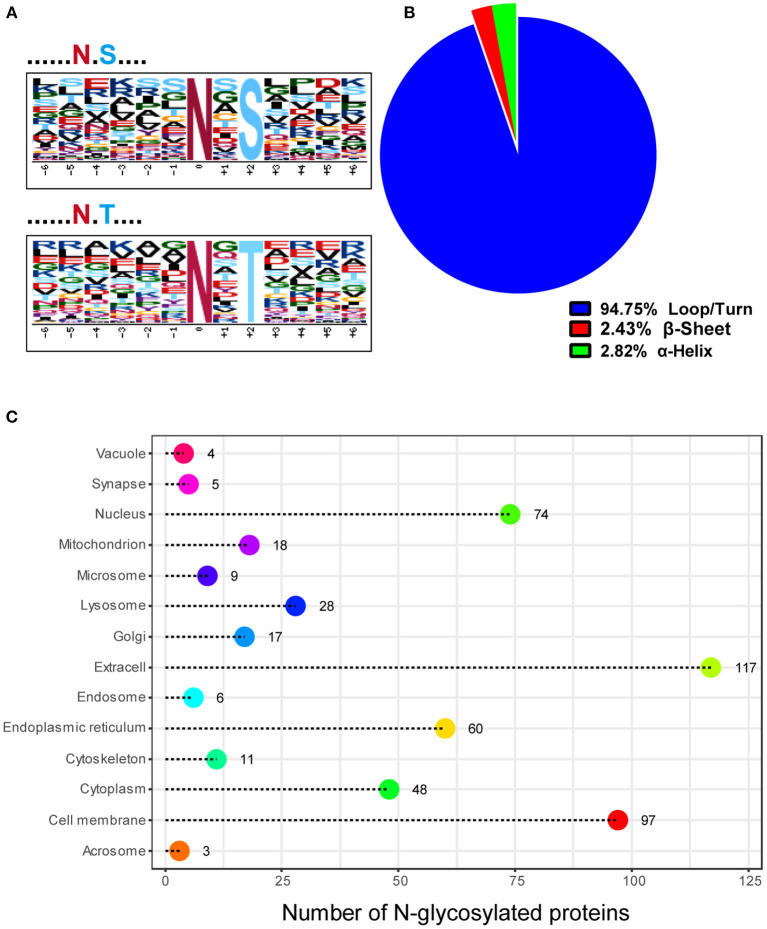
Characteristics of the N-glycosylation sites and subcellular localization of N-glycoproteins in mink testis during spermatogenesis. **(A)** Two distinct motifs rich in N-glycosylated peptides, N-X-T (top) and N-X-S (bottom), were identified by Motif-X software. **(B)** The proportion of N-glycosylation sites with loops/turns, beta-sheets, and alpha-helix secondary structures. The secondary structure of most sites was loops/turns. **(C)** Subcellular localization of the N-glycoproteins identified in mink testis.

We used the web-based Motif-X, which offers a simple interface to extract statistically significant motifs from big data sets like MS/MS post-translational modification data and proteomes that have a common biological function, to characterize the N-glycosylation sites discovered in this work. Except proline, all of the N-glycosylation sites matched the well-known Asn-X-Ser/Thr motif, according to our analyses (Figure 3A). Precisely, 62.56% (254 sites) of the N-glycosylation sites matched the Asn-X-Thr motif and 37.44% (152 sites) matched the Asn-X-Ser motif, which is consistent with an earlier study where more efficient N-glycosylation of the beta-asparagine was observed in the Asn-X-Thr motif compared to the Asn-X-Ser motif due to the higher affinity for the oligosaccharyltransferase (OST) active site (15, 16). Since glycosylation influences protein folding, protein solubility, antigenicity, biological activity, half-life, as well as cell-cell interactions, we further explored the relationship between N-glycosylation and the secondary structures of glycopeptides using DTU Bioinformatics online software. Our data showed that 94.75, 2.4, and 2.8% of the 533 glycosylation sites occurred in the loop/turn, β sheet, and α-helix structure, respectively (Figure 3B). In addition, the N-glycosylation modification plays a critical role in the localization and trafficking of proteins. Since proteins with N-glycosylation modifications are targeted to the secretory pathway, we predicted the signal peptide sequence and the subcellular localization of N-glycoproteins identified in our study through DTU Bioinformatics online software SignalP 4.1. Our analysis revealed that 185 (51.82%) of the 357 N-glycoproteins contained a signal peptide at the N-terminus (Supplementary Table S3). TMHMM Server v. 2.0, a program for predicting transmembrane helices based on a hidden Markov model (17) analysis showed that 175 (49.02%) N-glycoproteins contained transmembrane domains (Supplementary Table S3). Combining the SignalP 4.1 and TMHMM Server v. 2.0 analyses of N-glycoproteins identified in our study, 24.65% (88 out of 357) of proteins have neither a signal peptide nor a transmembrane domain. To further determine if these identified N-glycosylated proteins have the expected subcellular localization, we used Euk-mPLoc 2.0 (http://www.csbio.sjtu.edu.cn/bioinf/Cell-PLoc-2/), which combines many protein localization prediction methods, to predict their subcellular localization. The traditional secretory system was expected to receive 291 N-glycoproteins (81.51%), including those predicted to reside in the extracellular space (117), cell membrane (97), endoplasmic reticulum (60), Golgi apparatus (17), and vacuole (4) (Figure 3C).

### Quantitative analysis of N-glycoproteome in mink testes during seasonal spermatogenesis

**Figure 4:**
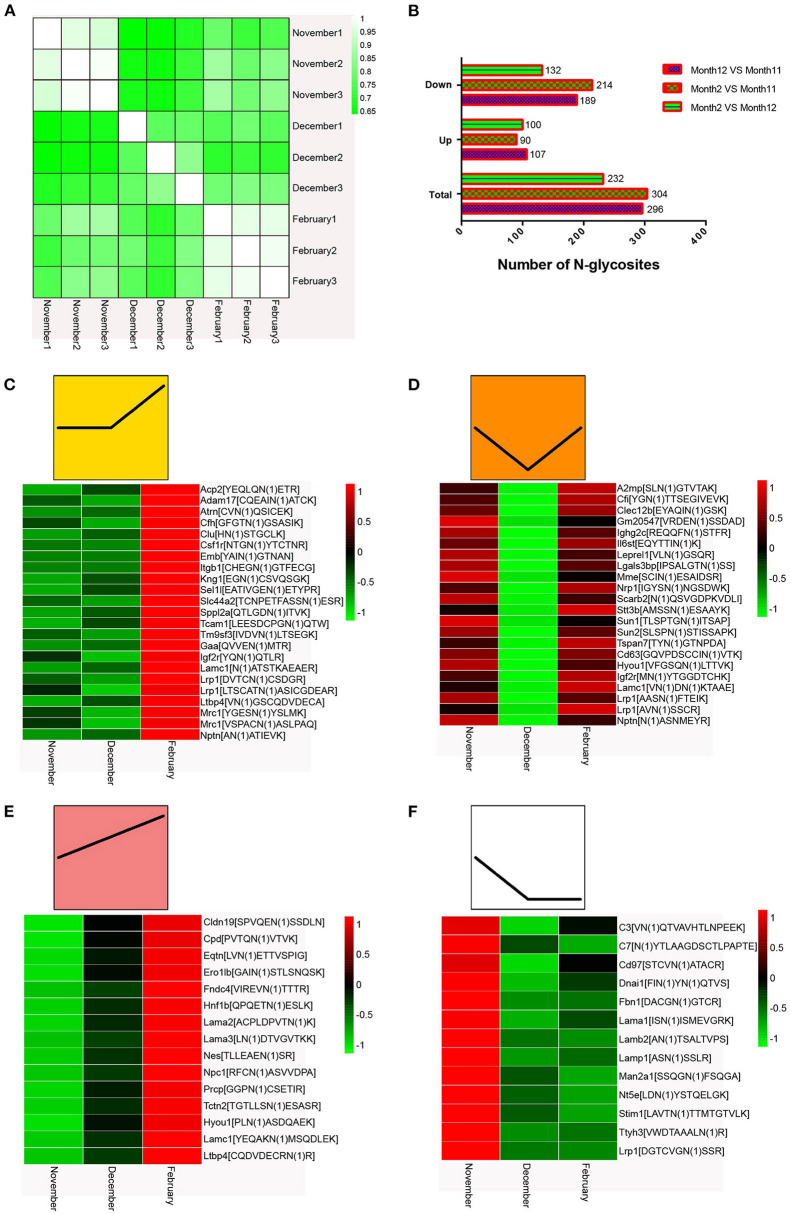
**(A)** Quantified repeatability of three biological repeats. **(B)** Changes in N-glycosidic peptides at different times after normalization of protein abundance. **(C–F)** Heat map of N-glycosylation sites in mink testis proteins (*p* < 0.05). Bar color represent a logarithmic scale from −1 to 1. Each column indicates a group and each row represents an N-glycosylation site.

To evaluate the quality and accuracy of the mink testis N-glycoproteome data determined by LC-MS/MS in this study, we investigated the reproducibility of N-glycoprotein expression levels in three different mink testis samples over the same period. All testis samples showed high correlation (*r* = 0.874–0.985) in three biological replicates of the same period (Figure 4A), indicating outstanding repeatability of the LC-MS/MS (LTQ-Orbitrap) study. Supplementary Figure 1 demonstrated the total number of glycosylation sites and glycoproteins detected and their overlap across biological replicates. The majority of N-glycoproteins detected in 9 samples (59.45–72.89%) were detected in each sample at different stages (Supplementary Figure 1). Therefore, the quantification results among the three replicates indicated reliable and reproducible data. In comparison to November samples, 296 and 304 proteins were differentially N-glycosylated in December and February samples (*P* < 0.05), respectively (Figure 4B and Supplementary Table S4). In addition, 232 N-glycosylated proteins were differentially modified in samples between December and February (Figure 4B and Supplementary Table S4).

The heat maps of the N-glycoproteome (N-glycoside-containing peptides and proteins) are shown in Figures 4C–F. We classified all the differentially modified N-glycosylated proteins into four model profiles. Notably, all significantly altered N-glycosylated proteins had a higher than 2-fold change. Among these, the differentially modified N-glycosylated proteins that were up-regulated in testes from December to February were involved in spermiogenesis (Figure 4C). Some differentially modified N-glycosylated proteins that were down-regulated in testes from November to December and subsequently up-regulated from December to February may promote spermatogonia renewal, the meiotic process, and an inhibitory effect on spermiogenesis (Figure 4D). The differentially modified N-glycosylated proteins that were consistently up-regulated in testes from November to February enhanced the testis development and spermatogenesis (Figure 4E). Those N-glycosylated proteins that were down-regulated in testes from November to December may be involved in the regulation of suppressing spermatogenesis (Figure 4F).

### Functional analysis of testicular proteins with different N-glycosylation during spermatogenesis in mink

**Figure 5:**
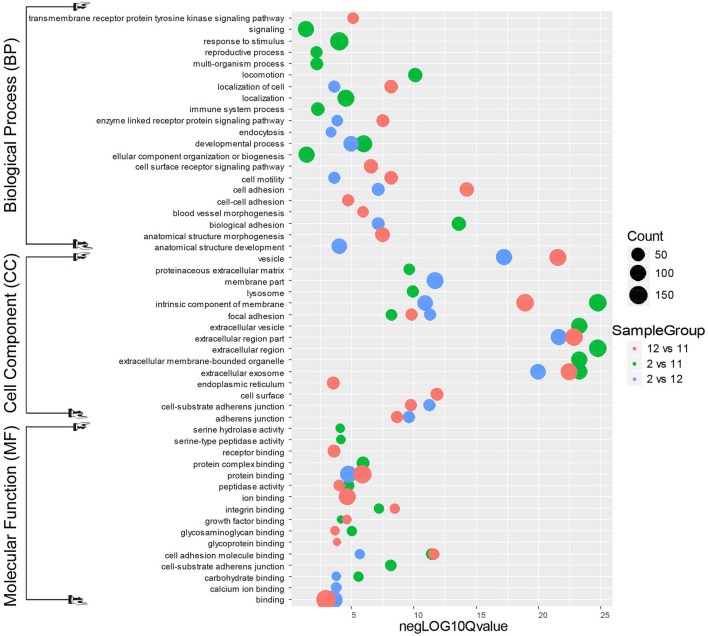
Gene Ontology analysis for differentially expressed mink testicular N-glycoproteins at different stages of spermatogenesis. All differentially expressed N-glycoproteins were clustered into the biological process (BP), cellular component (CC) and molecular function (MF) GO categories based on GO slim terms. Terms that were significantly enriched for N-glycoproteins (adjusted *P* < 0.05) are shown.

To further understand the biological roles of the differentially N-glycosylated proteins during the active spermatogenic phase in mink, we performed the Gene Ontology (GO) function term and the Kyoto Encyclopedia of Genes and Genomes (KEGG) pathway analysis. The *P*-value cut-off was 0.01, and *P*-values ordered the same category terms. Figure 5 shows an overview of the GO analysis with significantly enriched terms in the Biological Process (BP), Cell Component (CC), and Molecular Function (MF) categories.

In the biological process category of December mink testes vs. November mink testes (Figure 5), the N-glycoproteins with significant change were highly enriched in the processes of cell adhesion, cell motility, anatomical structure morphogenesis, and blood vessel morphogenesis. It can be inferred that these differential N-glycoproteins may play a vital role in the development of the blood-testis barrier (BTB). In the biological process category of February mink testes vs. December mink testes, the N-glycoproteins with significant change were enriched considerably in the processes of biological adhesion, developmental process, apoptotic cell clearance, and endocytosis. Nearly more than half of the differentiating spermatogonia undergo programmed cell death, namely, apoptosis. To provide energy to Sertoli cells and stop the release of hazardous contents, these dying cells and their leftover bodies must be swiftly phagocytosed and destroyed by Sertoli cells. Therefore, the differentially expressed N-glycoproteins associated with the biological processes mentioned above may contribute considerably to the spermatogenesis process. Differentially modified N-glycosylated proteins between February mink testes and November mink testes were significantly enriched in biological adhesion, developmental and reproductive processes.

As shown in Figure 5, in the cell component category of the most enriched differentially expressed N-glycoproteins from different groups (December vs. November, February vs. November, and February vs. December) were associated with intrinsic components of cell membranes, extracellular exosomes and vesicles, which suggests that these N-glycosylated proteins may play an important role in intracellular and extracellular communication. In the molecular function category of the most enriched differentially expressed N-glycoproteins from different groups (December vs. November, February vs. November, and February vs. December) was involved in cellular events such as binding, protein binding, ion binding, and cell adhesion molecule binding, which indicates that these glycosylated proteins may function in promoting various physiological and biochemical reactions in cells.

**Figure 6:**
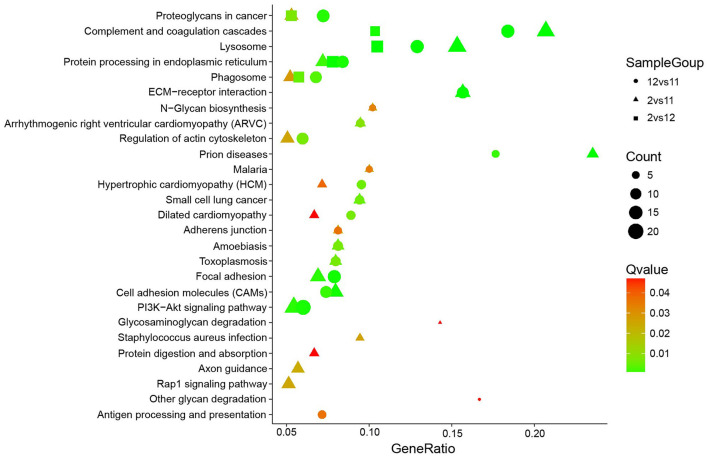
KEGG pathway analysis for differentially expressed mink testicular N-glycoproteins at different stages. The y-axis represents the name of the pathways.

KEGG pathway analysis of these altered N-glycoproteins in different groups (December vs. November, February vs. November, and February vs. December; Figure 6) showed that they were involved in PI3K-Akt signaling pathway, complement, and coagulation cascades, lysosome, focal adhesion, proteoglycans in cancer, protein processing in the endoplasmic reticulum, ECM-receptor interaction, cell adhesion molecules (CAMs), phagosome, and regulation of actin cytoskeleton. One interesting observation was a significant increase in phagosome changes. In our study 13 N-glycoproteins of the phagosome were significantly altered in the different groups (December vs. November, February vs. November, and February vs. December), including the integrin subunit alpha V (ITGAV), integrin subunit beta 1 (ITGB1), integrin subunit beta 2 (ITGB2), lysosome-associated membrane protein 2 (LAMP2), mannose receptor (MRC1), gamma-actin (Actg1), lysosomal-associated membrane protein 1 (LAMP-1), complement component 3 (C3), cation-dependent mannose-6-phosphate receptor (M6PR), cathepsin L (CTSL), macrophage scavenger receptor 1 (MSR1), transporter associated with antigen processing 1 (TAP1), and tubulin alpha-3a chain (TUBA3A). Strikingly, rapid phagocytosis of apoptotic cells by Sertoli cells contributes to spermatogonia maturation and maintenance of testicular homeostasis (18). These N-glycosylated proteins may play an active role in accelerating the efficiency of Sertoli cells to clear apoptotic cells from mink testes.

### Protein-protein interaction analysis of testicular proteins with various N-glycosylation modifications during spermatogenesis in mink

**Figure 7:**
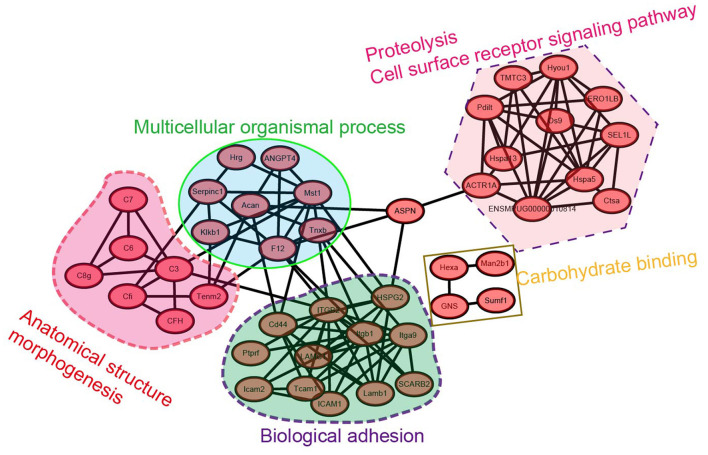
Protein-protein interaction network analysis for the N-glycoproteome of mink testis during seasonal spermatogenesis.

To identify the hub regulatory genes in N-glycosylation modification of testicular proteins, we explored the interaction of these differentially modified proteins using the STRING (Search Tool for the Retrieval of Interacting Genes/Proteins) interaction database (Figure 7). The names of the changed N-glycoproteins were represented on the protein-protein interaction (PPI) map by the names or locus numbers of the homologous proteins in *Mustela putorius furo*.

The largest subnet in the PPI network is composed of twelve biological adhesion-related N-glycoproteins, including LAMC1, Tcam1, ICAM1, ICAM2, integrin beta 1, and integrin alpha-9, etc. These biological adhesion-related N-glycoproteins are crucial to modulating the BTB function, suggesting that the synthesis of these N-glycoproteins is essential for budding or modulating the BTB. Moreover, eleven cell-surface receptor signaling pathways and proteolysis-related N-glycoproteins formed another subnet. The other large subnet was composed of four N-glycoproteins involved in carbohydrate binding, such as Man2b1, Hexa, GNS, and Sumf1. These network analysis-based findings tentatively suggest a functional relationship of differentially modified N-glycoproteins in mink testis in seasonal spermatogenesis.

### Gene transcription levels in mink testis at different stages of spermatogenesis were not correlated with protein glycosylation intensity

We chose 18 genes with differentially modified N-glycoproteins at various stages, such as SUN1, SUN2, Dnai1, Eqtn, Lama3, Jam2, Clu, and stim1 containing one N-glycosylation site, as well as MRC1, ERO1LB, CD63, Nptn, LTBP4, and LRP1 possessing multiple N-glycosylation sites, for detection of mRNA levels in the samples at different stages. As shown in Figures 8A,B, there was no necessary link between the mRNA levels and the corresponding protein glycosylation intensity of the genes mentioned above, suggesting that it is likely that N-glycosylation itself alters protein function, rather than protein level.

**Figure 8A:**
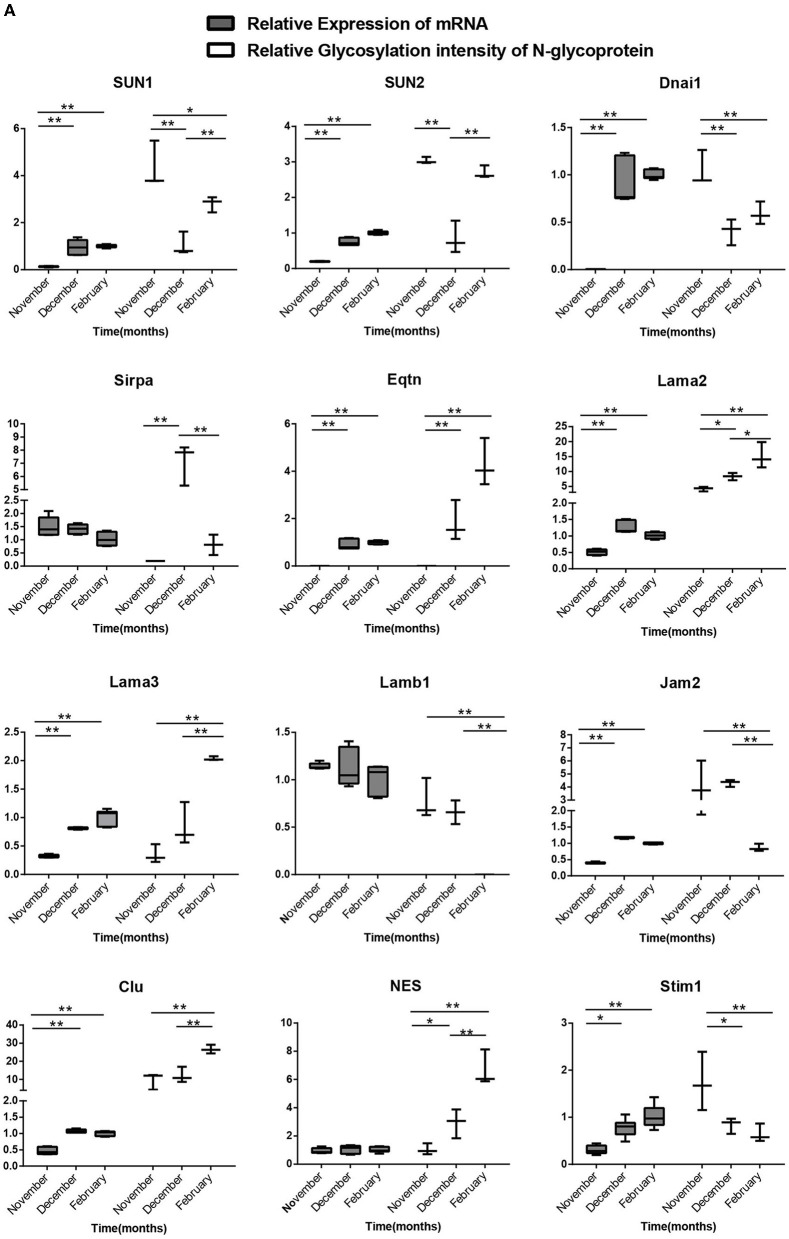
Relationship between the transcription levels of different N-glycosylated protein genes in different stages of spermatogenesis and the relative ionic strength of different N-glycosylated peptides in the corresponding proteins. One-way ANOVA with Tukey *post hoc* test was used for data statistics. Data are presented as mean with 95% CI. *P* < 0.05 was considered significant. *p < 0.05, **p < 0.01.

**Figure 8B:**
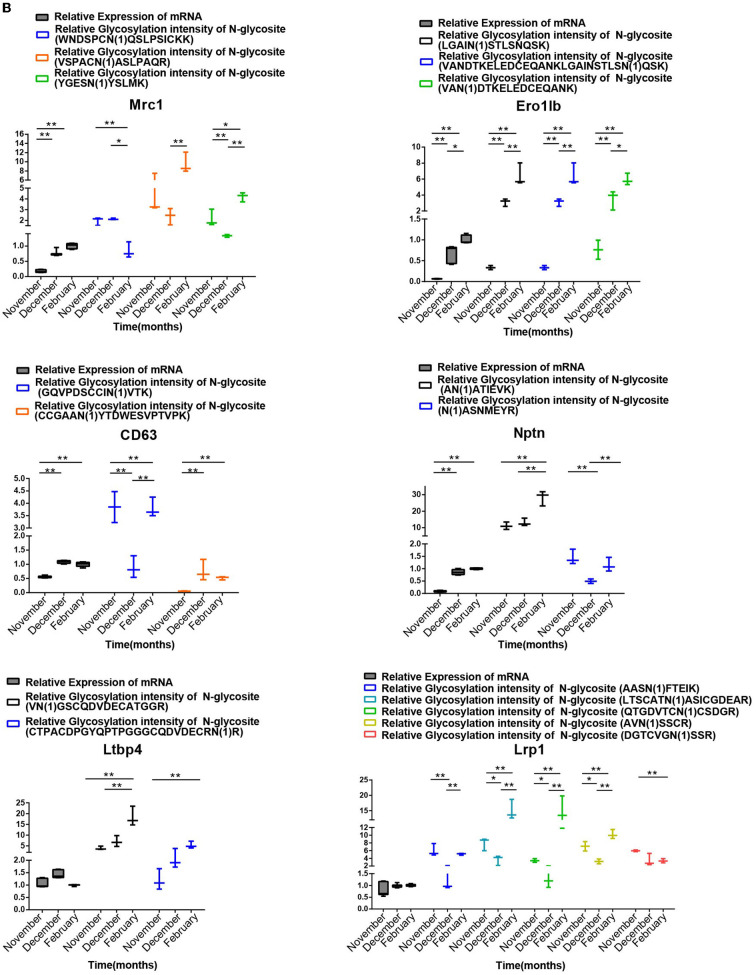
Analysis of the relationship between the transcript levels of different N-glycosylated testis protein genes in different stages of spermatogenesis and their relative ionic intensity of different N-glycosylated peptides contained in the corresponding proteins. One-way ANOVA with Tukey *post hoc* test was used for data statistics. Data are presented as mean with 95% CI. *P* < 0.05 was considered significant. *p < 0.05, **p < 0.01.

## Discussion

In this study, N-glycopeptide enrichment combined with Q-Exactive identification technology was used for the first time to identify N-glycosylation sites and glycosylated proteins in mink testis to determine the effect of N-glycoprotein modification on mink spermatogenesis. To generate a universal tag for MS analysis, the enriched peptides were treated with PNGase F in ${\text{H}}_{2}^{18}$O to remove N-glycans, convert asparagine (Asn) to heavy-oxygen aspartic acid (Asp) and cause a mass shift of + 2.9890 Da for glycosylation site identification by MS (18–20). This strategy is similar to previous proteolytic stable isotope labeling technique in which trypsin-digested peptides and individual proteins were labeled with heavy-oxygen water for the quantitative analysis of two proteome samples (19, 21). Based on the aforementioned technique, quantitative N-glycoproteomic analysis of mink testicular proteins at various spermatogenic stages identified 548 distinct N-glycopeptides, corresponding to 357 proteins with 532 non-redundant N-glycosylation sites. These non-redundant N-glycosylation sites are matched with the N-glycosylation consensus sequence motif (Asn-X-Ser/Thr; X≠ Proline). Interestingly, we found that low-density lipoprotein receptor-related protein 1 (LRP1) had 17 N-glycosides in this study. It is well known that LRP1 is a single-pass type-I membrane protein and is involved in the endocytosis and phagocytosis of apoptotic cells. It regulates cellular events, such as neuronal calcium signaling, neurotransmission, and kinase-dependent intracellular signaling (22). Many studies showed differentially glycosylated LRP1 in different tissues, and the proteolytic processing rate of LRP1 is dependent on the glycosylation state (23–26).

It is noteworthy that this study provides a changing landscape of quantitative N-glycoproteomic levels in recrudescent and active phase testis. Sertoli cells restore their structure and proliferation during the recrudescent phase, resulting in full-size testicles. BTB is formed through Sertoli-Sertoli cell junctions, which cause compartmentalization (26). Concomitantly, spermatogonia proliferate by mitosis resulting in increased numbers (27). Therefore, cell division is critical in recrudescent and active state testis. The main difference between the active phase and the recrudescent phase is the presence of mature sperm in the lumen of the seminiferous tubule at the active phase. Differentially altered N-glycoproteins identified in the testis during mink spermatogenesis are significantly enriched in the process of bioadhesion, reproductive processes and glycoprotein biosynthesis basing on GO category enrichment analysis.

Glycosylation regulates enzymatic processing by attaching glycans to peptide backbones to influence enzymatic activities (28, 29). In this study, we identified several enzymes as glycoproteins in mink testis. For instance, early gamete interactions in invertebrates and vertebrates, such as man, are mediated by molecules on the surface of sperm called beta-N-acetylhexosaminidases (HEXA) (30). In a previous report, the seminiferous epithelium of Hexa^−/−^ mice was comparable to that of wild-type mice in appearance, and topographical arrangement of its cell types at all ages examined (31). However, the epididymides of the mutant mice displayed obvious anomalies. These defects were mostly confined to the initial segment and intermediate zone (31). O-GlcNAc transferase (OGT) is an enzyme that adds O-GlcNAc to the serine/threonine residues of proteins from the donor substrate UDP-glucosamine (32). In our study, we identified OGT as an N-glycoprotein with two N-glycosites. This O-GlcNAcylation-producing enzyme is present in the epididymis, immature caput sperm, and maturing sperm (32). Consequently, N-glycosylation may influence OGT activity to regulate O-GlcNAcylation.

The differentially-glycosylated N-glycoproteins involved in reproductive processes, TAM receptor tyrosine kinases (Mertk), decorin (DCN), DEAD (Asp-Glu-Ala-Asp) box polypeptide 20 (DDX20), the selenoprotein P (SEPP1), hepatocyte nuclear factor 1-β (Hnf1b), arylsulfatase A (ARSA), cell adhesion molecule 1 (CADM1), A disintegrin and A metalloprotease 3 (ADAM3), cation sperm channel (Catsperd), equator (Eqtn) and others, was profound in spermatogenesis. For instance, Mertk knockout [Mertk (-/-)] mice had impaired testicular immune homeostasis. During development after sexual-maturity onset, germ cells progressively degenerate (33). In addition, decorin (DCN), a component of the extracellular matrix of the interstitial region and peritubular wall of the human testis, interacts with the growth factor to inhibit its biological function (34), and may also be involved in the regulation of paracrine signaling in human testes (34, 35). A coordinated series of mitotic and meiotic divisions, intricate cytodifferentiation steps, and dynamic intercellular interactions are all part of the fascinating and exquisitely complex process of spermatogenesis, which takes 6 to 9 weeks to complete. These processes are all controlled by the interaction of autocrine, paracrine, and endocrine factors. Therefore, we speculate that these differentially glycosylated N-glycoproteins identified in this study may play a crucial role in cell division and proliferation in mink testes.

Testicular BTBs are tight junctions between adjacent Sertoli cells and are located in the basal region of the seminiferous epithelium. Spermatogenesis relies on BTBs, which quarantine germ cells in the testis from the immune system to avoid being recognized as foreign and killed by immune cells (36, 37). The differentially glycosylated N-glycoproteins involved in the BTB formation, such as intercellular adhesion molecule-1 (Icam1), intercellular adhesion molecule-2 (Icam2), integrin subunit alpha E (Itgae), integrin subunit alpha X (Itgax), integrin subunit beta 1 (Itgb1), integrin subunit beta 2 (Itgb2), laminin gamma 1 (Lamc1), testicular cell adhesion molecule 1 (Tcam1), epithelial cell adhesion molecule (Epcam), a disintegrin and metallopeptidase domain 17 (Adam17), activated leukocyte cell adhesion molecule (Alcam), cadherin 2 (Cdh2), and claudin 19 (Cldn19), were identified in mink testes during spermatogenesis. Thus, these N-glycoproteins may affect seasonal spermatogenesis in mink by regulating the function of BTBs.

## Data availability statement

The datasets presented in this study can be found in online repositories. The names of the repository/repositories and accession number(s) can be found below: ProteomeXchange. The accession number is IPX0004884000.

## Ethics statement

The animal study was reviewed and approved by the Institute of Special Animal and Plant Sciences Committee on the Use of Live Animals in Teaching and Research.

## Author contributions

BX and YZ conceived and designed the experiments, performed the experiments, analyzed the data, contributed reagents, materials, analysis tools, and wrote the paper. Both authors contributed to the article and approved the submitted version.

## Funding

This work was supported by the Innovative research project in the field of experimental animals of Jilin Province (20210506022ZP), and the Science and Technology Innovation Program of the Chinese Academy of Agricultural Sciences (CAAS-ASTIP-2015-ISAPS).

## Conflict of interest

The authors declare that the research was conducted in the absence of any commercial or financial relationships that could be construed as a potential conflict of interest.

## Publisher's note

All claims expressed in this article are solely those of the authors and do not necessarily represent those of their affiliated organizations, or those of the publisher, the editors and the reviewers. Any product that may be evaluated in this article, or claim that may be made by its manufacturer, is not guaranteed or endorsed by the publisher.
