# Supplementary material for: Involvement of testicular N-glycoproteome heterogeneity in seasonal spermatogenesis of the American mink (Neovison vison)
Source: Front Vet Sci. 2022 Nov 4;9:1001431. doi: 10.3389/fvets.2022.1001431 (PMC9672844; doi:10.3389/fvets.2022.1001431)
Supplement: Supplementary file 1 [file Table_1.docx]

Table S1 Primer sequences and real-time PCR amplification parameters

| Genes | Accession No. | Primer (5'-3') | Product size/bp | Annealing temperature/℃ |
| --- | --- | --- | --- | --- |
| CD63 | XM_004773043.2 | GGACTGATTGCTGTGGGTGT  CCCACTGCGATGATGACGAT | 104 | 60.0 |
| CLU | XM_004775110.2 | TTAACCTGCACAGACTGCCC  GTGACGGATCTCCTTGCACA | 86 | 60.0 |
| DNAI1 | XM_004747428.2 | TCCAGCAAGTTCCTCGACAC  ACTGCCGCAAACACAGTAGA | 218 | 60.0 |
| EQTN | XM_004761778.2 | AATGCTGGGCATCTCGTTGA  GCTCTGGGTTGACGGTGTAT | 134 | 60.0 |
| ERO1LB | XM_022499173.1 | TGAACCCAGAGCGTTACACC  CACAAACCTTCCAGCCATGTG | 175 | 60.0 |
| JAM2 | XM_004757438.2 | TCCTAGTGGCTCCAGCAGTT  GGGCGGGATTCCCTTCTTTG | 102 | 60.0 |
| LAMA2 | XM_013061205.1 | TACGTTCACGGGCTGTATGG  GATGTTGGGGTACCAGCGAA | 101 | 60.0 |
| LAMA3 | XM_004742844.2 | CGGGGAGGCACTTATGTGTT  CCATCACACAGAGACCGCTT | 112 | 60.0 |
| LAMB1 | XM_004739520.2 | GGCAATCCCTCGGATATGGG  CTGCACCGTCCCCAAGTAAT | 234 | 60.0 |
| LRP1 | XM_004773175.2 | AGTGTGATGAGCGTACCTGC  TGGCACACGAGAACTCACTC | 162 | 60.0 |
| LTBP4 | XM_013047642.1 | CCCTTCGACATGCCTGACTT  TCGGCTTCCAGTCCCTCATA | 197 | 60.0 |
| MRC1 | XM_013058894.1 | AGGTTCACTAACTGGGCTGC  AAGGAATCCATGCCGTGTGT | 199 | 60.0 |
| NES | XM_013048944.1 | GGCCAAAGGGAGAGAACCC  AAAGGACCCTGGGAGTCTTG | 192 | 60.0 |
| NPTN | XM_013059368.1 | TCAACCGGGCAGAGTCTTTC  CCAAGGTGAGCCGGGTTATT | 120 | 60.0 |
| SIRPA | XM_004772926.2 | AGCCGGAGCATCTTCATTGT  GGTGTTCTTCTCGGGCTCAT | 147 | 60.0 |
| STIM1 | XM_004768190.2 | CTTTCAGTTCTGAAGGCCACG  CTGTCAGAAGGGGGTGGGTC | 223 | 60.0 |
| SUN1 | XM_013047769.1 | TGTCGCAACCACGATGAGAA  CTTCAAGTGCTGCCCTACCA | 135 | 60.0 |
| SUN2 | XM_004743259.2 | GGACGCTTGTCACTTCTCCA  ATGAGAAGCGCCTGGTCAAA | 126 | 60.0 |
| GAPDH | NM_001310173.1 | GTATGATTCCACCCACGGTA  CACCCCATTTGATGTTGGCG | 122 | 60.0 |
