# Supplementary figures and images for: Involvement of testicular N-glycoproteome heterogeneity in seasonal spermatogenesis of the American mink (Neovison vison)
Source: Front Vet Sci. 2022 Nov 4;9:1001431. doi: 10.3389/fvets.2022.1001431 (PMC9672844; doi:10.3389/fvets.2022.1001431)

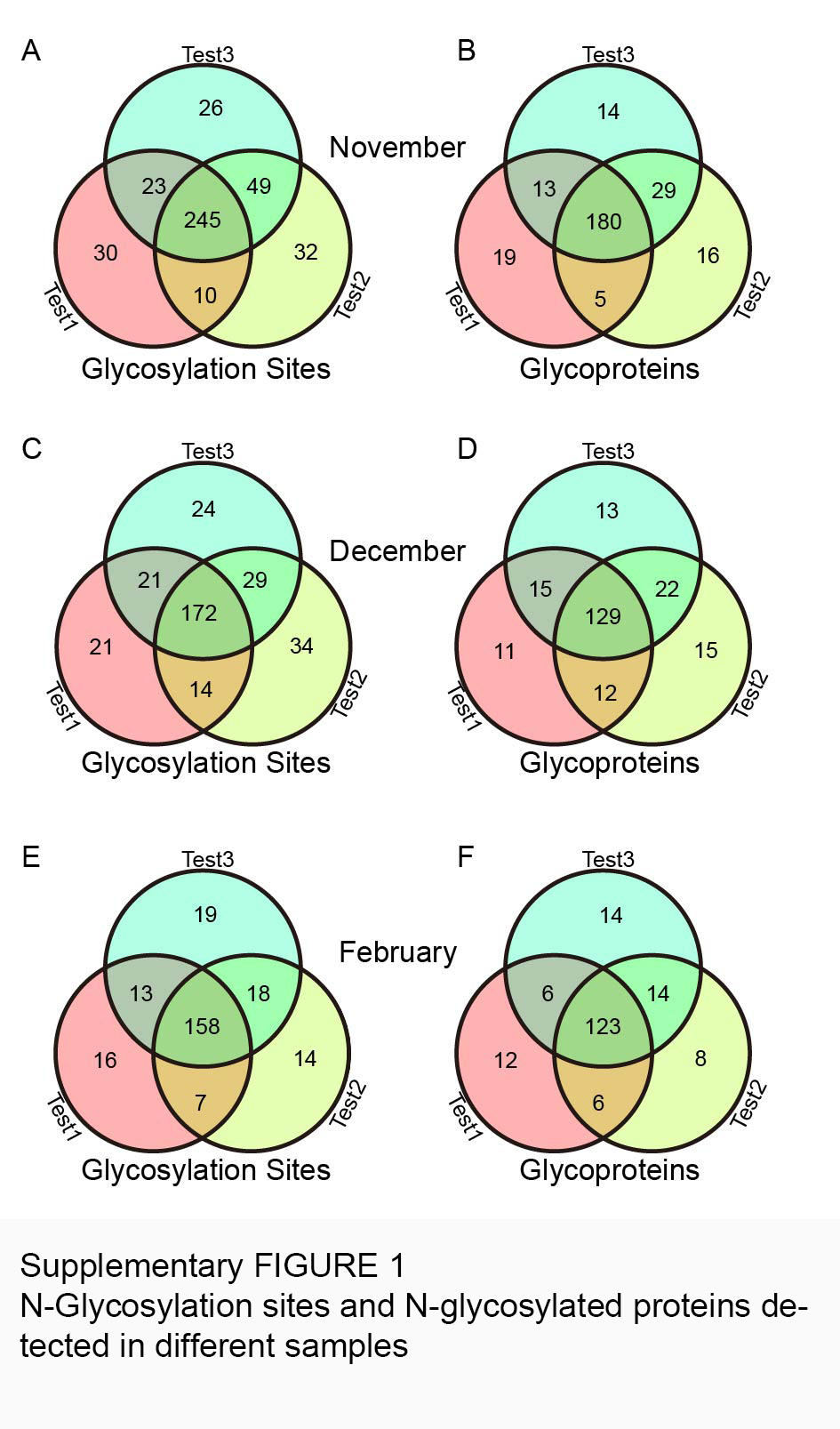

Supplement: Supplementary file 5 [file Image_1.tif]
